# The Feasibility of a Guideline-Directed Medical Therapy Rapid Up-Titration Programme Among Real-World Heart Failure Patients: A Multicentre Observational Study

**DOI:** 10.3390/jcm14103611

**Published:** 2025-05-21

**Authors:** Fanni Bánfi-Bacsárdi, Arnold Péter Ráduly, Attila Borbély, Noémi Nyolczas, Attila Szilágyi, Tamás G. Gergely, Zsolt Forrai, Judit Papp, Orsolya Rátosi, Tünde Rácz, Krisztina Hati, Ildikó Kocsis, Zoltán Csanádi, Gábor Zoltán Duray, Péter Andréka, Zsolt Piróth, Balázs Muk

**Affiliations:** 1 Department of Adult Cardiology, Gottsegen National Cardiovascular Center, 1096 Budapest, Hungary; fanni.banfi-bacsardi@gokvi.hu (F.B.-B.);; 2 Doctoral School of Clinical Medicine, University of Szeged, 6720 Szeged, Hungary; 3 Division of Cardiology, Department of Cardiology, Faculty of Medicine, University of Debrecen, 4032 Debrecen, Hungary; 4 Division of Clinical Physiology, Department of Cardiology, Faculty of Medicine, University of Debrecen, 4032 Debrecen, Hungary; 5 Kálmán Laki Doctoral School, University of Debrecen, 4032 Debrecen, Hungary; 6 Department of Cardiology, Central Hospital of Northern Pest-Military Hospital, 1134 Budapest, Hungary; 7 Markhot Ferenc Teaching Hospital and Clinic, 3300 Eger, Hungary; 8 Erzsébet Teaching Hospital and Rehabilitation Institute Sopron, 9400 Sopron, Hungary; 9 Heart and Vascular Center, Semmelweis University, 1122 Budapest, Hungary

**Keywords:** rapid up-titration programme, heart failure, guideline-directed medical therapy, heart failure outpatient clinic

## Abstract

**Background**: The 2023 ESC Heart Failure (HF) Guidelines recommend the rapid up-titration of guideline-directed medical therapy (GDMT) for all patients after HF hospitalisation. Real-world data on the implementation of a rapid up-titration programme (RTP) are scarce. **Methods:** We aimed to summarise the primary experiences of a six-week RTP in a multicentre observational study of five cardiology centres, evaluating the GDMT applied and the target doses (TDs) achieved during the RTP. The safety of RTP in relation to exceeding the “safety indicators” used in the STRONG-HF trial and any serious adverse events were observed. Changes in the left ventricular ejection fraction (LVEF) after RTP were evaluated. **Results:** Among the 90 consecutive patients (age: 56 [49–63] years, HFrEF: 96%, NT-proBNP at discharge: 1390 [735–2835] pg/mL; continuous variables are presented as median and interquartile ranges, while categorical variables are shown as absolute numbers and percentages, respectively), a remarkable proportion of patients received GDMT at hospital discharge; however, target doses were rarely achieved (RASi: 100%, TD RASi: 11%; βB: 97%, TD βB: 6%; MRA: 99%, TD MRA: 82%; SGLT2i: 98%, TD SGLT2i: 98%; triple therapy [TT: RASi + βB + MRA]: 96%, TD TT: 2%, quadruple therapy [QT: RASi + βB + MRA]: 94%, TD QT: 2%). After the six-week RTP, 100% of the total cohort (TC) were receiving RASi; 99–99–99% were receiving βB, MRA, and SGLT2i medications; and altogether, 98–98% were on TT and QT. In total, 78–78% of the patients received ≥50% of the TDs of TT and QT, while 51–51% of the TC were on TDs of TT and QT. During the RTP, no serious adverse events were observed. Between two and four months after the RTP, 51% of HFrEF patients evolved to the HFimpEF category. **Conclusions:** The present multicentre, observational study confirms that RTP is feasible and safe in real-world clinical practice, leading to a remarkably large proportion of patients receiving GDMT by the end of the six-week RTP, resulting in a significant increase in LVEF.

## 1. Introduction

According to the PREVEND study, the lifetime risk of heart failure (HF) remains high, as nearly one in four individuals develops HF [1]. Across the whole spectrum of HF, clinicians must insist on the application of prognosis-modifying guideline-directed medical therapy (GDMT) [2,3,4]. The literature suggests that the use of first-line quadruple therapy (QT: RASi [renin-angiotensin system inhibitor]: angiotensin-converting enzyme inhibitor [ACEi]/angiotensin receptor blocker [ARB]/angiotensin receptor neprilysin inhibitor [ARNI] + beta-blocker [βB] + mineralocorticoid receptor antagonist [MRA] + sodium-glucose co-transporter 2 inhibitor [SGLT2i]) could prevent nearly 1.2 million deaths in the HF with reduced ejection fraction (HFrEF) patient population over one year [5].

The early prognostic benefit of the introduction of GDMT has been well-established for both neurohormonal antagonists and SGLT2is in landmark trials [6,7,8,9,10,11]. According to these data, “time to GDMT” has come under the spotlight, as described in terms of acute coronary syndrome as “door to balloon” time [12,13]. It is well-known that the early post-discharge phase is a highly vulnerable period associated with a high morbidity and mortality burden [14,15].

Without a doubt, the STRONG-HF trial, published in 2022, led to a paradigm shift and can be declared a practice-changer in the therapy optimisation routine of HF [16]. In the STRONG-HF trial, 1078 patients hospitalised for acute HF were randomised into “high-intensity care” and “usual care” groups: after hospital discharge, the former group of patients participated in the rapid up-titration programme (RTP) with closer follow-up (FUP), while the latter group received conventional therapy optimisation as part of the standard of care. Among those randomised to the RTP, GDMT optimisation was projected to be completed within several weeks post-discharge. In the trial, specific safety indicators were established to ensure the safety and efficacy of the RTP. With the guidance of these safety indicators, RTP performed within several weeks after discharge resulted in a significantly lower combined risk of all-cause mortality or readmission to hospital for HF within six months compared to conventional treatment (adjusted risk difference = 8.1%; 95% confidence interval = 2.9–13.2, *p* = 0.0021; risk ratio = 0.66; 95% confidence interval = 0.50–0.86). In light of the STRONG-HF trial, the 2023 Focused Update of the 2021 European Society of Cardiology (ESC) HF Guidelines (GLs) recommends the initiation and rapid up-titration of GDMT for all patients after HF hospitalisation within six weeks to reduce the risk of HF rehospitalisation or death (Class I recommendation, level of evidence: B) [3]. However, the STRONG-HF trial on which the recommendation was based applied strict randomisation criteria suggesting that the broad, universal, safe feasibility of RTP may cause difficulties in daily clinical practice, leading to a major challenge for cardiologists and HF specialists in the near future [17,18].

Real-world data on the implementation of RTP in everyday practice are scarce. In accordance with the 2023 ESC HF GLs, a six-week RTP was designed and implemented in the daily clinical practice of the HF Outpatient Clinics (HFOCs) of five secondary/tertiary cardiology centres in Hungary.

We aimed to summarise our primary experiences within the framework of a multicentre observational study, evaluating:

- The GDMT applied and target doses (TDs) achieved during the RTP;
- The safety of RTP considering the fulfilment of the “safety indicators” used in the STRONG-HF trial [16] and the occurrence of serious adverse events;
- The effect of the occurrence of the safety indicators, frailty syndrome, and presence of multimorbidity and HF categories on the success of RTP;
- Changes in the quality of life (QoL) of patients;
- Changes in the value of the left ventricular ejection fraction (LVEF) due to the effect of the RTP.

## 2. Materials and Methods

### 2.1. Study Population and Design

A multicentre, retrospective, observational study was conducted after the publication of the 2023 ESC HF GLs at five central European (Hungarian) secondary/tertiary cardiology centres. Patients hospitalised for HF between 1 January 2024 and 31 October 2024 and who did not receive optimal medical therapy at discharge according to current ESC HF GLs [2,3] were considered eligible for RTP based on the four-parameter model established using the main randomisation criteria of the STRONG-HF trial (systolic blood pressure [SBP] ≥ 100 mm Hg + heart rate [HR] ≥ 60 min^−1^ + serum potassium ≤ 5 mmol/L + estimated glomerular filtration rate [eGFR] ≥ 30 mL/min/1.73 m^2^) [18]. HF and its LVEF-based categories (HFrEF, HF with mildly reduced ejection fraction [HFmrEF], and HF with preserved ejection fraction [HFpEF]) were defined according to the 2021 Universal Definition of HF [19]. In accordance with the STRONG-HF trial and the 2023 Focused Update of the 2021 ESC HF GLs [3,16], the whole spectrum of HF patients was included in the RTP (i.e., HFrEF, HFmrEF, and HFpEF categories). Participation in the RTP was voluntary.

**Figure 1:**
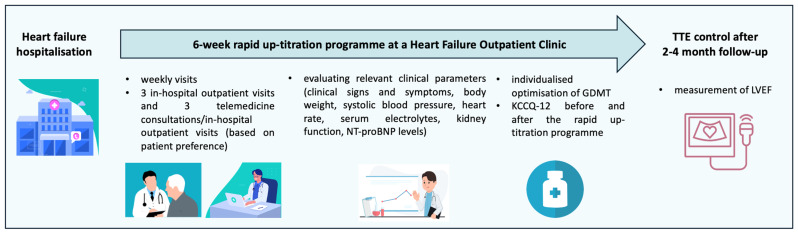
Structure of the rapid up-titration programme. KCCQ-12: 12-item Kansas City Cardiomyopathy Questionnaire; GDMT: guideline-directed medical therapy; LVEF: left ventricular ejection fraction; NT-proBNP: N-terminal pro-B type natriuretic peptide; TTE: transthoracic echocardiography.

A six-week, potentially universal RTP was designed and implemented in the daily clinical practice of the HFOCs of five cardiac centres (Figure 1). The six-week RTP consisted of six weekly visits: three outpatient in-hospital visits on even weeks and three telemedicine/outpatient in-hospital visits (based on patient preference) on odd weeks. During the FUP visits, relevant clinical parameters (including clinical signs and symptoms, body weight, SBP, HR, serum electrolytes, kidney function, and N-terminal pro-B type natriuretic peptide [NT-proBNP] levels) were determined. ECGs were performed during in-hospital outpatient visits. Taking all these parameters into account, therapy optimisation during the six-week RTP was individualised. The TDs of each medication were defined as stated in the ESC 2021 HF GLs [2].

During the RTP, we also examined the fulfilment of the “safety indicators” that guided the continuation or discontinuation of therapy optimisation in the STRONG-HF trial (SBP < 95 mm Hg, HR < 55 min^−1^, serum potassium > 5 mmol/L, eGFR < 30 mL/min/1.73 m^2^, >10% increase in NT-proBNP compared to NT-proBNP at discharge). We assessed the efficacy and safety of the RTP among those individuals whose parameters did or did not exceed the safety indicators.

Changes in patients’ QoL were measured before RTP and at the end of RTP using the 12-item Kansas City Cardiomyopathy Questionnaire (KCCQ-12) [20,21] as part of the routine standard of care.

Serious adverse events during the six-week RTP and the FUP period were observed, defined as the occurrence of rehospitalisation or death.

After finishing the six-week RTP, 2–4-month FUP data on the value of LVEF were obtained using transthoracic echocardiography findings.

The presence of the frailty syndrome in patients was also evaluated in line with the four domains (clinical domain [comorbidities, weight loss, falls], psycho-cognitive domain [cognitive impairment, dementia, depression], functional domain [impairment of activities of daily living and instrumental activities of daily living, low/non-mobility, balance], and social domain [living alone, no social support, institutionalisation]) suggested in the position paper published by the HF Association (HFA) of the ESC in 2019 [22].

The occurrence of non-cardiovascular comorbidities (NCCMs) and their effect on GDMT optimisation were investigated. Ten NCCMs were evaluated: obesity (body mass index [BMI] > 30 kg/m^2^), Type I or Type II diabetes mellitus (DM), eGFR measured in hospital < 60 mL/min/1.73 m^2^), hyperuricaemia, hypo- or hyperthyroidism, sleep-disordered breathing, asthma/chronic obstructive pulmonary disease (COPD), anaemia (haemoglobin measured in hospital < 130 g/L in men and <120 g/L in women), iron deficiency (ferritin < 100 μg/L or transferrin saturation [TSAT] < 20%), and dyslipidaemia.

Our study protocol was reviewed and approved by the National Scientific and Ethical Committee of Hungary (approval number: BM/22646-1/2024), and the present study adheres to the ethical principles of the Declaration of Helsinki (1964) and its later amendments [23].

### 2.2. Statistical Analysis

Clinical data were obtained from the participating hospitals’ patient management systems. Data were documented in anonymised form in a Microsoft Excel 16.80 spreadsheet (Microsoft Corporation, Redmond, WA, USA), and IBM SPSS Statistics 26.0 (International Business Machines Corporation, Armonk, NY, USA) was used for the statistical calculations.

The distribution of continuous variables was tested with the Shapiro–Wilk normality test. Continuous variables are presented as median and interquartile ranges (considering their non-Gaussian distribution), while categorical variables are shown as absolute numbers and percentages.

The results of the KCCQ-12 and main haemodynamic and laboratory parameters were compared before the RTP and at the end of RTP, while in-hospital LVEF values and 2–4-month control LVEF parameters were compared using the Wilcoxon test. Subgroup analyses were performed with Fisher’s exact test to compare the proportion of patients achieving TT, QT, and TDs of TT and QT between patients meeting 0–1 vs. 2–4 frailty domain criteria between HFrEF and non-HFrEF subgroups, between patients with <3 NCCMs and with ≥3 NCCMs, and between patients fulfilling and not fulfilling the safety indicators. Six-week TDs of QT were compared using the Chi-square test considering the fulfilment of each safety indicator.

Statistical significance was defined as *p* < 0.05.

## 3. Results

The data of 90 consecutive HF patients requiring hospitalisation due to HF who participated in the RTP were analysed. Eighty-two per cent of patients were male, with a median age of 56 [49–63] years. The median LVEF measured in-hospital before the RTP was 24 [20–32]%. At baseline, 96% of patients belonged to the HFrEF category, 1% to the HFmrEF category, and 3% to the HFpEF category. Sixty-three per cent of the cohort were newly diagnosed HF patients (“de novo” HF).

The severity and complexity of the patient cohort were represented by the high rate of comorbidities and an elevated median NT-proBNP value at admission. The baseline characteristics of the total cohort are presented in Table 1.

At hospital admission, 54% of patients were treated with RASi (ACEi/ARB: 52% [TD ACEi/ARB: 21%], ARNI: 2% [TD ARNI: 0%]), 48% with βB (TD βB: 7%), 38% with MRA (TD MRA: 12%), and 23% with SGLT2i medications (TD SGLT2i: 23%). In total, 28% were on triple therapy (TT: RASi + βB + MRA; TD TT: 2%), while 17% had QT (TD QT: 1%). One per cent of patients had cardiac resynchronisation therapy with or without a defibrillator (CRT-P or CRT-D) at admission, while 4% had undergone previous implantable cardioverter defibrillator (ICD; without CRT) implantation (Table 1).

At hospital discharge, a remarkable proportion of patients received GDMT; however, TDs were rarely achieved (RASi: 100%, TD RASi: 11%; βB: 97%, TD βB: 6%; MRA: 99%, TD MRA: 82%; SGLT2i: 98%, TD SGLT2i: 98%; TT: 96%, TD TT: 2%, QT: 94%, TD QT: 2%). At hospital discharge, 99% of the cohort were on loop diuretics (with a median dose of 40 [40–70] mg furosemide equivalents) and 7% were on thiazide diuretics (Table 1).

**Figure 2:**
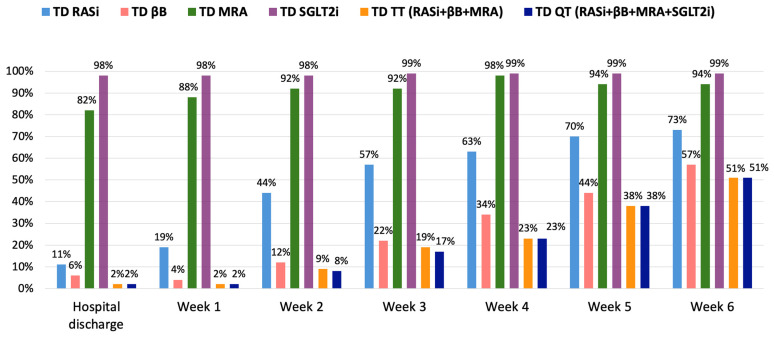
Proportion of patients on TDs of GDMT during the six-week RTP. GDMT: guideline-directed medical therapy; MRA: mineralocorticoid receptor antagonist; QT: quadruple therapy; RASi: renin-angiotensin system inhibitor; SGLT2i: sodium-glucose co-transporter 2 inhibitor; TD: target dose; TT: triple therapy; βB: beta-blocker.

**Figure 3:**
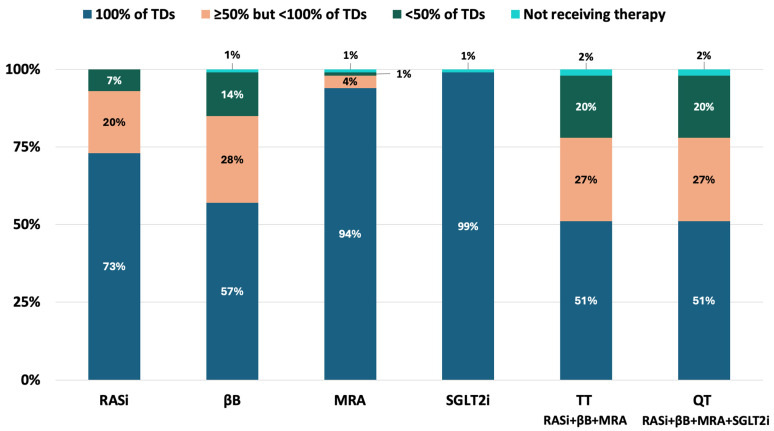
Doses of GDMT achieved at the end of the six-week RTP. GDMT: guideline-directed medical therapy; MRA: mineralocorticoid receptor antagonist; QT: quadruple therapy; RASi: renin-angiotensin system inhibitor; SGLT2i: sodium-glucose co-transporter 2 inhibitor; TD: target dose; TT: triple therapy; βB: beta-blocker.

After the six-week RTP, RASi was applied in 100% of patients, and βB, MRA, and SGLT2i in 99% of patients each. Altogether, 98–98% of patients were treated with TT and QT. TDs of RASis were achieved in 73% of the total cohort, TDs of βBs in 57%, TDs of MRAs in 94%, and TDs of SGLT2i in 99% (Figure 2). Further, 78% of patients received ≥ 50% of the TDs of TT, and 78% received ≥ 50% of the TDs of QT (Figure 3). TDs of TT and QT were achieved in 51–51% of the cohort after the six-week RTP. At the end of the RTP, 100% of patients had loop diuretics (with a median dose of 40 [38–80] mg furosemide equivalents), while 14% were being treated with thiazide diuretics.

**Figure 4:**
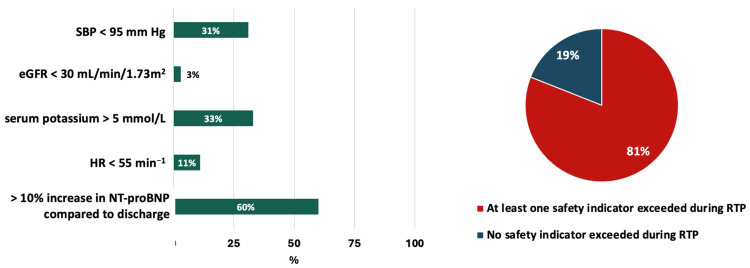
Fulfilment of the safety indicators during the six-week RTP. eGFR: estimated glomerular filtration rate; HR: heart rate; NT-proBNP: N-terminal pro-B type natriuretic peptide; RTP: rapid up-titration programme; SBP: systolic blood pressure.

During the RTP, the safety indicator for SBP was achieved in 31% of cases, for HR in 11%, for eGFR in 3%, and for serum potassium in 33%. NT-proBNP levels increased by more than 10% compared to the discharge values in 60% of cases (Figure 4). In 19% of patients, RTP was accomplished without exceeding any safety indicators, while in 38% of the cohort, neither SBP, HR, serum potassium, nor the eGFR safety indicators were exceeded, except for the threshold for NT-proBNP.

TDs of TT and TDs of QT achieved at the end of the six-week RTP were not influenced by the fulfilment of the safety indicators for serum potassium, HR, and NT-proBNP. Patients exceeding the safety indicator for eGFR were less likely to be treated with TT and QT. However, the use of TT and QT with TDs did not differ according to this safety indicator. Exceeding the safety indicator for SBP resulted in significantly lower use of TT and QT with TDs (Supplementary Materials, Table S1); however, among the patients exceeding the safety indicator for SBP, the proportion of patients on TDs of TT and QT remained remarkably high.

There were no serious adverse events during RTP or FUP, and the RTP was found to be safe. Comparing the main characteristics of the cohort at baseline (at hospital discharge) and at the end of the RTP, the value of median SBP, eGFR, and serum potassium levels did not change in the effect of GDMT optimisation (Supplementary Materials, Table S2).

From the beginning to the end of the RTP period, we observed a significant increase in both the overall summary score (34 [26–45] vs. 57 [51–62] points; before vs. after RTP) and the clinical summary score for KCCQ-12 (27 [19–34] vs. 44 [40–47] points; before vs. after RTP) (*p* < 0.001) (Supplementary Materials, Figure S1). KCCQ-12 scores did not differ between patients achieving and not achieving TDs of QT at the end of the RTP (overall summary score (57 [54–62] vs. 56 [48–61] points; achieving vs. not achieving TDs of QT after RTP) and the clinical summary score for KCCQ-12 (44 [41–47] vs. 43 [37–47] points; achieving vs. not achieving TDs of QT after RTP).

Considering the domains of frailty syndrome, according to the ESC HFA position paper published in 2019, 98% of the cohort fulfilled the clinical domain, 11% the psycho-cognitive domain, 8% the functional domain, and 6% the social domain. All four domains were simultaneously observed in 2% of the patients, while 16% of the total cohort were associated with 2–4 frailty domains.

The subgroup analysis incorporating the domains of frailty syndrome revealed that the proportion of patients on TT or QT was slightly lower among those associated with 2–4 domains at the end of the RTP, although the use of TDs of TT and QT did not differ significantly among them.

An increase in the burden of comorbidities (≥3 NCCMs), the chronic HF categories, and exceeding any safety indicators did not influence the achieved TDs of GDMT at the end of the six-week RTP (Supplementary Materials, Figure S2).

**Figure 5:**
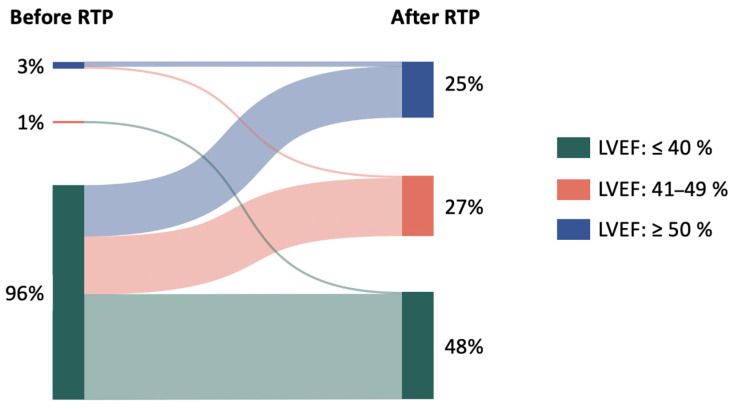
Changes in LVEF in the effect of the RTP. LVEF: left ventricular ejection fraction; RTP: rapid up-titration programme.

Two to four months after completing the RTP, during FUP, the median LVEF had increased from 24 [20–32]% to 41 [30–49]% (*p* < 0.001). Sixty-two per cent of patients had a ≥10% increase in LVEF; altogether, 51% of HFrEF patients evolved to the HF with improved ejection fraction (HFimpEF) category (Figure 5).

In the effect of the RTP, the proportion of non-HFrEF patients significantly increased (4% vs. 52%, *p* < 0.05) (Supplementary Materials, Figure S3).

## 4. Discussion

### 4.1. Main Findings

Our multicentre real-world observational analysis suggests that RTP is feasible in daily practice and revealed that TDs of GDMT were achieved in a significant proportion of the real-world patients participating in the RTP. After careful patient selection according to the main randomisation criteria of the STRONG-HF trial, no serious adverse events occurred during RTP. Thus, RTP was considered safe with close FUP.

According to our data, GDMT was implemented and successfully up-titrated with the guidance of the fulfilment of safety indicators, including SBP, HR, kidney function, serum potassium, and NT-proBNP, as observed in the STRONG-HF trial. The presence of frailty syndrome negatively influenced the implementation of GDMT during RTP. However, among those patients with an increased burden of comorbidities and who fulfilled the frailty criteria described in the ESC HFA position paper, high doses of TT and QT could be implemented as well. During a short FUP period, the median value of LVEF increased significantly by a median of 17% due to the effect of the implementation of GDMT during the RTP. Moreover, 51% of the HFrEF patients were re-classified to the HFimpEF category.

Finally, due to the effect of the RTP, QoL measured using KCCQ-12 improved significantly.

### 4.2. GDMT and Its Rapid Up-Titration in HF Patients

Despite the clear, robust clinical data [9,24,25] and the current GLs [2,3,26], the use of the strategic first-line GDMT in everyday clinical practice remains suboptimal [27,28,29,30,31]. A recent analysis from the Get With The Guidelines-HF Registry for the period between 2021 and 2023 showed that the prevalence of TT was 35.2%, 23.5% for SGLT2i, and 13% for QT [32]. A European retrospective observational study for a similar examination period revealed that the use of SGLT2is (19% vs. 60%; admission vs. discharge) and QT (19% vs. 54%; admission vs. discharge) significantly increased in a patient cohort hospitalised for HF after the publication of the 2021 ESC HF GLs, which underscores the feasibility of implementing the recommendations of the international GLs into real-world clinical practice [2,33,34].

Since the publication of the 2021 ESC HF GLs, several studies have examined possible strategies for therapy optimisation and the potential benefits of RTP [35,36,37]. The first randomised controlled trial (RCT) to provide clinical evidence on the safety and efficacy of RTP was the STRONG-HF trial [16,38]. In addition, it should be emphasised that the incidence of serious adverse events was not significantly increased with RTP [16]. RTP was also shown to be more effective than the conventional therapy optimisation sequence, irrespective of sex, age, kidney function, the presence of atrial fibrillation/flutter, underlying HF risk profile, LVEF, SBP, and NT-proBNP, among other factors, based on secondary analyses of the STRONG-HF trial [39,40,41,42,43,44,45]. However, it is worth noting that the use of SGLT2is was relatively low because of the study period. It is also important to note that in the STRONG-HF trial, in addition to HFrEF patients, patients with HFmrEF and HFpEF were included; the latter two HF categories comprised more than one-third of the total cohort [16]. For these reasons, the 2023 ESC HF GLs do not differentiate among HF categories in terms of RTP; thus, RTP is recommended across the whole spectrum of HF [2,3].

To the best of our knowledge, prior to the 2023 ESC HF GLs, only a few studies on real-life experience with a limited number of patients regarding the implementation of RTP had been published, and no clear evidence has emerged since then on the feasibility of RTP in light of the updated GLs. The TEAM-HF trial was published by Spahillari et al. in 2024, in which 114 patients with LVEF < 50% participated in an RTP at a dedicated “GDMT Clinic” between 6 May 2021 and 30 September 2023, thus mainly before the publication of the 2023 ESC HF GLs, over a median of 15.8 weeks and a median of 4.5 visits [46]. It must be emphasised that only 2.6% of the participants were hospitalised for HF within a month of referral and they had a median NT-proBNP of 587 [138–960] ng/L, so compared to the STRONG-HF trial and the current study, a less complex HF patient group was probably examined.

Comparison of the GDMT achieved in the STRONG-HF trial, TEAM-HF trial, and the current analysis is difficult due to differences in the cohorts and RTP protocols (Supplementary Materials, Table S3) [16,46]. However, it should be underscored that the proportion of patients with a higher burden of comorbidities was very different in these trials.

While acknowledging the study’s limitations, the medical therapy and TDs achieved by the end of the up-titration process matched or exceeded those of the STRONG-HF and the TEAM-HF trial across relevant parameters (Supplementary Materials, Table S3). The application ratio of QT and TD QT registered in the TEAM-HF trial was lower than in the current analysis (QT: 88.0% vs. 98%, ≥50% TD QT: 44.0% vs. 78%, TD QT: 24.0% vs. 51%; TEAM-HF vs. current analysis).

### 4.3. Fulfilment of Safety Indicators During the RTP

There are a few reasons why therapy optimisation and achieving TDs of GDMT may be difficult during RTP [47,48,49]. Tomasoni et al. analysed the impact on the prognosis of the five safety indicators that most influenced the feasibility of RTP in the STRONG-HF trial [50]. Patients for whom at least one safety indicator was exceeded were less likely to achieve TDs than those who did not exceed any (mean difference: 11%; 95% confidence interval = −13.6–−8.4%; *p* < 0.001) [50]. Furthermore, it should also be emphasised that the composite endpoint of rehospitalisation due to HF and all-cause mortality did not differ between those who exceeded safety indicators and those who did not [50]. According to the literature, the use of SGLT2is and MRAs may be beneficial in patients with lower SBP, as they are associated with the least adverse SBP change [51,52]. The safe use of first-line medications in patients with low SBP may also result in an increase in SBP [53,54,55,56,57]. In light of this evidence, it is not surprising that among patients undergoing RTP, the early introduction and TD use of SGLT2is and MRAs were observed in the current analysis, facilitating easier optimisation of RASi and βB therapy.

In our study, the safety criterion for SBP was met in 31% of patients undergoing RTP, which exceeded the rate detected in the STRONG-HF trial (9.4%) but did not affect the proportion of patients on QT (QT: 96% vs. 98%; *p* = 0.538; those exceeding the safety indicator vs. not exceeding the safety indicator for SBP), while the TDs of QT achieved were negatively influenced by the significant decrease in SBP during the RTP (TD QT: 32% vs. 60%; *p* = 0.022; those exceeding the safety indicator vs. not exceeding the safety indicator for SBP). This phenomenon may be attributed to the distinct characteristics of the patient cohorts examined.

Among patients in the RTP group of the current analysis, the occurrence of the safety indicator for HR was moderately higher than in the STRONG-HF trial (8.5% vs. 11%; “high-intensity care group” of the STRONG-HF trial vs. current study), but the present analysis showed that this did not affect treatment optimisation (QT: 100% vs. 97%; *p* = 1.000; TD QT: 51% vs. 50%; *p* = 1.000; those exceeding the safety indicator vs. not exceeding the safety indicator for HR).

In the STRONG-HF trial, 15.5% of patients experienced a decline in eGFR ≥ 15%, but this was not associated with an adverse effect on the primary endpoint; however, early eGFR decline (≥15%) was associated with the administration of lower drug doses [40]. It is also noteworthy that the six-month prognosis was more favourable in the high-intensity care group, irrespective of baseline eGFR [40]. In our study, 3% of patients met the safety indicator for kidney dysfunction, a rate similar to that detected in the STRONG-HF trial (5.2%), which affected the application of QT (QT: 33% vs. 100%; *p* = 0.001; those exceeding the safety indicator vs. not exceeding the safety indicator for eGFR).

Hyperkalaemia is one of the main limiting factors of the up-titration to TDs of neurohormonal antagonist therapy. In the STRONG-HF trial, the safety indicator for elevated serum potassium levels was exceeded in 27.5% of patients during RTP [16]. In our analysis, serum potassium levels > 5 mmol/L were observed in 33% of patients during the six-week RTP. However, no permanent interruption of the RTP was needed in any of these patients, and the optimal treatment achieved did not differ significantly based on this safety threshold (QT: 100% vs. 97%; *p* = 0.545; TD QT: 57% vs. 48%; *p* = 0.505; those exceeding the safety indicator vs. not exceeding the safety indicator for serum potassium). The positive effect of novel pharmacotherapies (SGLT2i, ARNI) on reducing the risk of severe hyperkalaemic events [25,58] may facilitate the introduction and dose optimisation of other prognosis-modifying drugs [59,60,61].

The assessment of NT-proBNP levels may be of paramount importance in identifying patients with a poorer prognosis at hospital discharge [62]. A secondary analysis of the GUIDE-IT study showed that patients whose NT-proBNP levels decreased to ≤1000 pg/mL during therapy optimisation had a more favourable outcome [63]. In the STRONG-HF trial, it should be emphasised that elevated NT-proBNP levels during RTP did not prevent success [42], but RTP in these patients may undoubtedly require more caution. In line with these findings, delaying the initiation of strategic drug therapy or suspending therapy optimisation based solely on elevated NT-proBNP levels is not justified, as it could reduce the beneficial impact of first-line therapy on prognosis. In the current study, NT-proBNP levels were elevated by >10% compared with the values at discharge in 60% of patients, exceeding those seen in the STRONG-HF trial (41.1%) while still achieving a high rate of QT (QT: 96% vs. 100%; *p* = 0.513; TD QT: 46% vs. 57%; *p* = 0.384; those exceeding the safety indicator vs. not exceeding the safety indicator for NT-proBNP). During the six-week RTP, no significant change was observed in the median NT-proBNP value (1390 [735–2835] vs. 1459 [618–3241] pg/mL; before vs. after RTP).

### 4.4. The Effect of RTP on QoL

In the STRONG-HF trial, patients ranked their state of health on a EURO-QoL 5-Dimension (EQ-5D) questionnaire at baseline and at 90 days of FUP [64]. One of the strongest predictors of the greatest improvement in QoL was the high-intensity care treatment itself. However, it must be highlighted that the success of RTP and the achieved TDs were not dependent on the baseline level of QoL. Further, all patients with and without any safety indicators witnessed an increment in their QoL. However, the occurrence of a safety indicator was associated with a smaller improvement in QoL [50]. According to our analysis, the majority of the cohort showed a significant improvement in both the overall summary and the clinical summary score of the KCCQ-12.

### 4.5. Changes in LVEF Due to RTP

The implementation of modern GDMT can lead to reverse remodelling and improvement in left ventricular systolic dysfunction [63,65,66,67,68,69,70,71].

Based on recent meta-analyses and observational studies [72,73,74], 19.5–29% of HFrEF patients were reclassified as HFimpEF patients due to the effect of modern HF therapy. According to the analysis by Veltmann et al., at the end of a FUP period of almost one year, 77% of 598 real-world patients diagnosed with “de novo” HFrEF improved to LVEF > 35% after therapy optimisation (ACEi/ARB/ARNI + βB + MRA) [75].

In the TEAM-HF trial, an absolute 6% increase in LVEF (37 [31–41]% vs. 43 [38–53]%, *p* < 0.001, before vs. after RTP) was observed [46], which is exceeded by the results of the present study, which indicated a median 17% increase in LVEF (24 [20–32]% vs. 41 [30–49]%, *p* < 0.001, before vs. after RTP). However, comparing these results is challenging due to the differences in patient populations and study periods.

### 4.6. The Effect of Frailty Syndrome on GDMT Implementation During the RTP

According to the literature, the presence of frailty syndrome has an undoubtedly negative effect on the implementation of GDMT and prognosis. However, data regarding the efficacy of implementing modern GDMT for HF and RTP are scarce.

This unfavourable phenomenon was revealed in the GUIDE-IT trial as well, in which patients with a high frailty burden were associated with a lower likelihood of achieving GDMT (TT: 17.7% vs. 28.4%; high frailty vs. non-frail patients) and frailty was correlated with a higher risk of HF hospitalisation/mortality (hazard ratio = 1.76, 95% confidence interval = 1.20–2.58) [76]. However, in this analysis, the use of TDs of GDMT was not reported.

The proportion of patients fulfilling the frailty criteria outlined in the 2019 ESC HFA position paper was quite high. Moreover, according to our data, these patients were less likely to be treated with GDMT. Despite this, 86% of the patients fulfilling 2–4 frailty domains received TT, 86% had QT at the end of RTP, and the proportion of patients treated with TDs of TT (51%) and QT (51%) remained remarkably high in comparison to the data in the recent literature [16,28,46]. This may raise awareness of the possibility of the conscious application of pharmacotherapy and the importance of multidisciplinary outpatient care in the most vulnerable HF subgroup.

### 4.7. The Effect of Comorbidities on GDMT Implementation During the RTP

An increase in the number of comorbidities also negatively influences the implementation of GDMT [77]. The comorbidity burden of our study cohort was comparable to the recent data from large registries [78,79]. The 58% of patients having ≥3 NCCMs in our study exceeded the 11.4% revealed in the “high-intensity care” group of the STRONG-HF trial [16]. Even though the median age of the current patient cohort was notably younger than in the SwedeHF Registry (76 [67–82] years), the comorbidity profile was comparable [79]. Despite the high comorbidity burden, 96–96% of patients in the current study with ≥3 NCCMs were receiving TT and QT at the end of RTP, while TD TT and QT were being applied in 43–43%.

### 4.8. The Effect of the HF Category on GDMT Implementation During the RTP

Despite the lack of Class I evidence for the use of neurohormonal antagonists in HFmrEF and HFpEF, the full spectrum of HF categories was included in the STRONG-HF trial [16]. It is reasonable to state that among the majority of patients in these HF categories, due to co-existing comorbidities, a RASi, an MRA, or a βB is indicated. In our analysis, no differences were detected in the GDMT achieved in the HFrEF and non-HFrEF subgroups.

## 5. Conclusions

The present multicentre observational study confirms that RTP is feasible and safe in real-world clinical practice and leads to a remarkably large proportion of patients receiving prognosis-modifying GDMT. Our results also revealed a notable improvement in QoL at the end of a six-week up-titration period. With the guidance provided by the safety indicators applied in the STRONG-HF trial, pharmacotherapy was successfully implemented and optimised in the majority of the examined cohort. A remarkable proportion of patients (62%) experienced a ≥10% increase in LVEF. Due to the effect of the RTP among those patients suffering from HFrEF, a large proportion (51%) of them improved to the HFimpEF category.

### Limitations

The analysis included only patients considered eligible for RTP based on the four-parameter model [18], which increases potential selection bias. The real-world patient population of our multicentre study consisted exclusively of individuals of the Caucasian race, so our results and conclusions cannot be applied with certainty to those outside of this group. Participation in the RTP was voluntary. Despite the lack of Class I evidence for the use of neurohormonal antagonists in HFmrEF and HFpEF, the full spectrum of HF was included in our analysis, in accordance with the STRONG-HF trial and the 2023 ESC HF GLs [3,16]. Another limitation is the size of the cohort under study and the short FUP period. The size of the study population may also underpower the ability to detect serious safety events. The present analysis was not intended to identify the aetiology of HF.

## Figures and Tables

**Table 1:** Main characteristics of the cohort.

Parameters	Total Cohort (*n* = 90)
Male sex (%)	82
Age, median [IQR], years	56 [49–63]
De novo heart failure (%)	63
LVEF, median [IQR], %	24 [20–32]
HFrEF (%)	96
HFmrEF (%)	1
HFpEF (%)	3
Duration of hospitalisation, median [IQR], days	9 [7–13]
**Comorbidities**	
Coronary artery disease (%)	28
Hypertension (%)	68
Atrial fibrillation/flutter (%)	41
Stroke (%)	1
PAD (%)	2
Severe VHD (%)	7
Previously diagnosed chronic kidney disease (%)	8
eGFR < 60 mL/min/1.73 m^2^ (%)	28
Dyslipidaemia (%)	71
Iron deficiency (%)	68
Obesity (%)	66
Diabetes (%)	34
Hyperuricaemia (%)	23
Anaemia (%)	11
Hypo-/hyperthyroidism (%)	11
Asthma/COPD (%)	10
Sleep-disordered breathing (%)	3
≥3 NCCMs (%)	58
**At admission**	
Heart rate, median [IQR], min^−1^	97 [87–114]
Systolic blood pressure, median [IQR], mmHg	126 [114–140]
Serum creatinine, median [IQR], μmol/L	98 [85–115]
eGFR, median [IQR], mL/min/1.73 m^2^	71 [59–85]
Serum potassium, median [IQR], mmol/L	4.1 [3.8–4.5]
Serum sodium, median [IQR], mmol/L	139 [136–140]
Haemoglobin, median [IQR], g/L	150 [138–161]
NT-proBNP, median [IQR], pg/mL	4095 [2352–8160]
RASi (%)	54
ACEi/ARB (%)	52
ARNI (%)	2
βB (%)	48
MRA (%)	38
TT (%)	28
SGLT2i (%)	23
QT (%)	17
TD RASi (%) *	21
TD ACEi/ARB (%) *	21
TD ARNI (%) *	0
TD βB (%) *	7
TD MRA (%) *	12
TD TT (%) *	2
TD SGLT2i (%) *	23
TD QT (%) *	1
CRT-D/CRT-P (%)	1
ICD (without CRT-D) (%)	4
**At discharge**	
Heart rate, median [IQR], min^−1^	78 [70–85]
Systolic blood pressure, median [IQR], mmHg	112 [105–121]
Serum creatinine, median [IQR], μmol/L	103 [87–119]
eGFR, median [IQR], mL/min/1.73 m^2^	67 [55–83]
Serum potassium, median [IQR], mmol/L	4.4 [4.1–4.7]
Serum sodium, median [IQR], mmol/L	139 [136–141]
NT-proBNP, median [IQR], pg/mL	1390 [735–2835]
RASi (%)	100
ACEi/ARB (%)	69
ARNI (%)	31
βB (%)	97
MRA (%)	99
TT (%)	96
SGLT2i (%)	98
QT (%)	94
TD RASi (%) *	11
TD ACEi/ARB (%) *	11
TD ARNI (%) *	0
TD βB (%) *	6
TD MRA (%) *	82
TD TT (%) *	2
TD SGLT2i (%) *	98
TD QT (%) *	2
Loop diuretics (%)	99
Thiazide diuretics (%)	7

ACEi: angiotensin-converting enzyme inhibitor; ARB: angiotensin receptor blocker; ARNI: angiotensin receptor neprilysin inhibitor; COPD: chronic obstructive pulmonary disease; CRT-D/CRT-P: cardiac resynchronisation therapy with or without defibrillator; eGFR: estimated glomerular filtration rate; HFmrEF: heart failure with mildly reduced ejection fraction; HFpEF: heart failure with preserved ejection fraction; HFrEF: heart failure with reduced ejection fraction; ICD: implantable cardioverter defibrillator; IQR: interquartile range; LVEF: left ventricular ejection fraction; MRA: mineralocorticoid receptor antagonist; NCCM: non-cardiovascular comorbidity; NT-proBNP: N-terminal pro-B type natriuretic peptide; PAD: peripheral artery disease; QT: quadruple therapy; RASi: renin-angiotensin system inhibitor; SGLT2i: sodium-glucose co-transporter 2 inhibitor; TD: target dose; TT: triple therapy; VHD: valvular heart disease; βB: beta-blocker; *: the proportion of patients on target doses of each medication is relative to the total cohort.

## Data Availability

All data generated or analysed during this study are included in this article. Anonymised data from the study cohort can be provided upon reasonable request. The formal request must specify the exact purpose for which the data will be used, taking into account the relevant ethical standards. Enquiries can be directed to the corresponding author, Balázs Muk (e-mail: balazs.muk@gokvi.hu), who will forward the request to an independent review committee who are empowered to provide access. A data-sharing agreement must be signed prior to access. The anonymised data of the cohort will be provided in a secure data-sharing environment. Researchers whose proposed use of the data has been approved may access the data, which can be used only for the approved specific purposes.

## Supplementary Materials

The following supporting information can be downloaded at: https://www.mdpi.com/article/10.3390/jcm14103611/s1, Table S1. Effect of the fulfilment of the safety indicators for TD TT and QT application at the end of the six-week RTP; Table S2. Comparison of the main characteristics of the patient population at baseline and at the end of the six-week RTP; Table S3. Comparison of main characteristics and GDMT of patient cohorts in the STRONG-HF trial and the TEAM-HF trial; Figure S1. Results of KCCQ-12; Figure S2. Effect of the fulfilment of the frailty domains, HF categories, ≥3 NCCMs and safety indicators on the TD achieved at the end of the six-week RTP; Figure S3. Distribution of HF categories in the effect of the RTP.
