# Supplementary material for: The Feasibility of a Guideline-Directed Medical Therapy Rapid Up-Titration Programme Among Real-World Heart Failure Patients: A Multicentre Observational Study
Source: J Clin Med. 2025 May 21;14(10):3611. doi: 10.3390/jcm14103611 (PMC12112513; doi:10.3390/jcm14103611)
Supplement: Supplementary file 1 [file jcm-14-03611-s001.zip › jcm-3604155-supplementary.pdf]

Supplementary Materials

The feasibility of a guideline-directed medical therapy rapid up-titration programme among real-world heart failure patients: a multicentre observational study

Fanni Bánfi-Bacsárdi, Arnold Péter Ráduly, Attila Borbély, Noémi Nyolczas, Attila Szilágyi, Tamás G. Gergely, Zsolt Forrai, Judit Papp, Orsolya Rátosi, Tünde Rácz, Krisztina Hati, Ildikó Kocsis, Zoltán Csanádi, Gábor Zoltán Duray, Péter Andréka, Zsolt Piróth, Balázs Muk

Supplementary Materials, Table S1. Effect of the fulfilment of the safety indicators for TD TT and QT application at the end of the six-week RTP

	**Exceeded safety indicator for SBP**	**Did not exceed safety indicator for SBP**	***p-value***
TT, (%)	96%	98%	0.538
**TD TT, (%)**	**32%**	**60%**	**0.022**
QT, (%)	96%	98%	0.538
**TD QT, (%)**	**32%**	**60%**	**0.022**
	**Exceeded safety indicator for eGFR**	**Did not exceed safety indicator for eGFR**	***p-value***
**TT, (%)**	**33%**	**100%**	**0.001**
TD TT, (%)	0%	53%	0.112
**QT, (%)**	**33%**	**100%**	**0.001**
TD QT, (%)	0%	53%	0.112
	**Exceeded safety indicator for serum potassium**	**Did not exceed safety indicator for serum potassium**	***p-value***
TT, (%)	100%	97%	0.545
TD TT, (%)	57%	48%	0.505
QT, (%)	100%	97%	0.545
TD QT, (%)	57%	48%	0.505
	**Exceeded safety indicator for HR**	**Did not exceed safety indicator for HR**	***p-value***
TT, (%)	100%	97%	1.000
TD TT, (%)	51%	50%	1.000
QT, (%)	100%	97%	1.000
TD QT, (%)	51%	50%	1.000

	**Exceeded safety indicator for NT-proBNP**	**Did not exceed safety indicator for NT-proBNP**	***p-value***
TT, (%)	96%	100%	0.513
TD TT, (%)	46%	57%	0.384
QT, (%)	96%	100%	0.513
TD QT, (%)	46%	57%	0.384
	**Exceeded any safety indicator**	**Did not exceed any safety indicator**	***p-value***
TT, (%)	97%	100%	1.000
TD TT, (%)	50%	56%	0.784
QT, (%)	97%	100%	1.000
TD QT, (%)	50%	56%	0.784

eGFR: estimated glomerular filtration rate; HR: heart rate; NT-proBNP: N-terminal pro-B type natriuretic peptide; QT: quadruple therapy; RTP: rapid up-titration programme; SBP: systolic blood pressure; TD: target dose; TT: triple therapy.

Supplementary Materials, Table S2. Comparison of the main characteristics of the patient population at baseline and at the end of the six-week RTP

Parameters	At baseline (at hospital discharge)	At the end of the RTP	*p-value*
Heart rate, median [IQR], min^{-1}	78 [70-85]	69 [64-78]	<0.001
Systolic blood pressure, median [IQR], mmHg	112 [105-121]	108 [99-120]	0.124
Serum creatinine, median [IQR], µmol/L	103 [87-119]	104 [89-123]	0.395
eGFR, median [IQR], $mL/min/1.73m^2$	67 [55-83]	69 [52-85]	0.215
Serum potassium, median [IQR], mmol/L	4.4 [4.1-4.7]	4.3 [4.1-4.7]	0.351
Serum sodium, median [IQR], mmol/L	139 [136-141]	138 [136-140]	0.214
NT-proBNP, median [IQR], pg/mL	1390 [735-2835]	955 [538-2290]	0.050

eGFR: estimated glomerular filtration rate; IQR: interquartile range; NT-proBNP: N-terminal pro-B type natriuretic peptide; RTP: rapid up-titration programme.

Supplementary Materials, Figure S1. Results of KCCQ-12

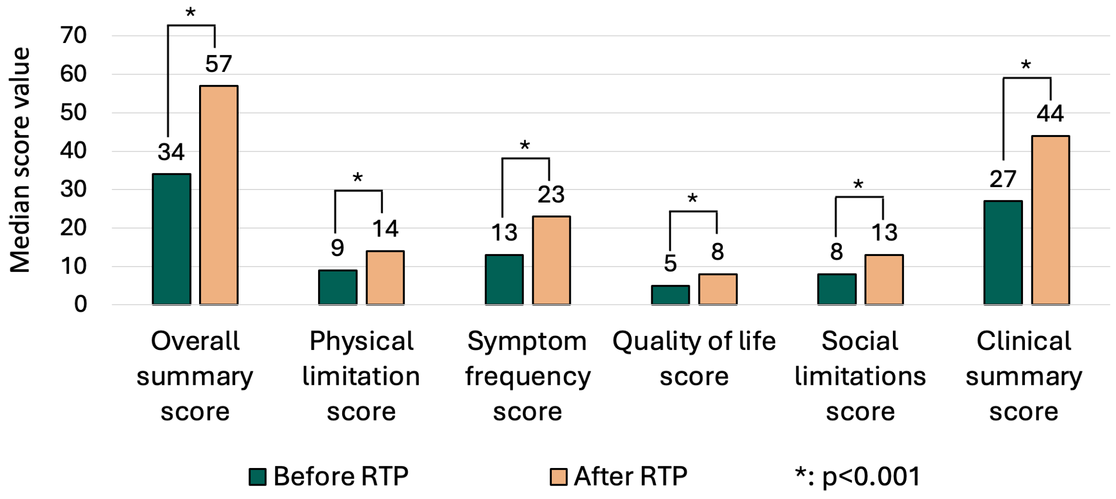

KCCQ: Kansas City Cardiomyopathy Questionnaire; RTP: rapid up-titration programme.

Supplementary Materials, Figure S2. Effect of the fulfilment of the frailty domains, HF categories, ≥ 3 NCCMs and safety indicators on the TD achieved at the end of the six-week RTP

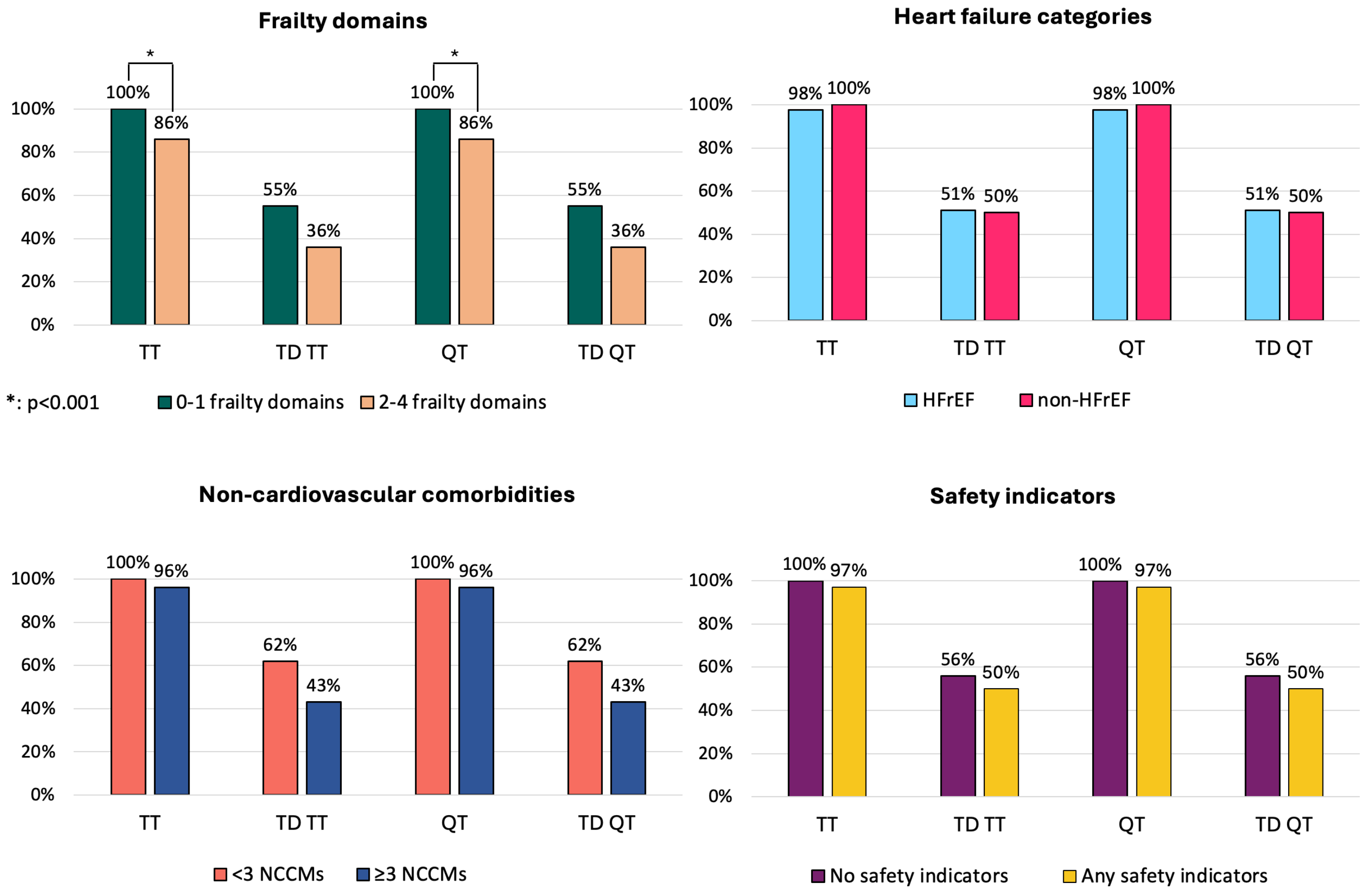

HFrEF: heart failure with reduced ejection fraction; NCCMs: non-cardiovascular comorbidities; QT: quadruple therapy; RTP: rapid up-titration; TD: target dose; TT: triple therapy.

Supplementary Materials, Figure S3. Distribution of HF categories in the effect of the RTP

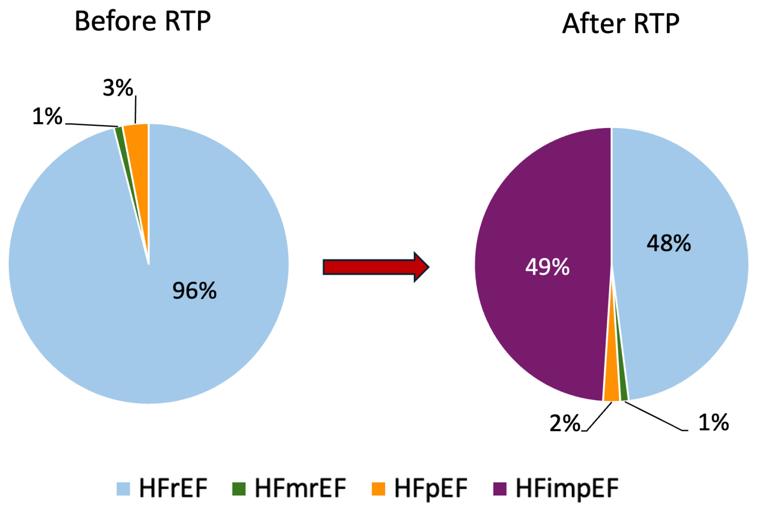

HFimpEF: heart failure with improved ejection fraction; HFmrEF: heart failure with mildly reduced ejection fraction; HFpEF: heart failure with preserved ejection fraction; HFrEF: heart failure with reduced ejection fraction; RTP: rapid up-titration programme.

Supplementary Materials, Table S3. Comparison of main characteristics and GDMT of patient cohorts in the STRONG-HF trial and the TEAM-HF trial

Parameters	STRONG-HF [16] "High-intensity care group" (n=542)	TEAM-HF [49] "GDMT Clinic" (n=114)	Current study (n=90)
Male sex, (%)	60	71.9	82
Age, mean ± SD/median [IQR], years	62.9 ± 13.5	67.6 ± 14.6	56 [49-63]
Previous hospitalisation primarily due to heart failure (before RTP), (%)	100	2.6 (within a month)	100
De novo heart failure, (%)	14	-	63
LVEF at baseline, mean ± SD/median [IQR], %	36.7 ± 12.57	37 [31-41]	24 [20-32]
HFrEF, (%)	67	72	96
HFmrEF, (%)	17	28	1
HFpEF, (%)	16	-	2
DM, (%)	28	22.8	34
Coronary artery disease, (%)	48	74.6	28
Hypertension, (%)	51	73.7	68
Atrial fibrillation/flutter, (%)	42	43.0	41
≥3 non-cardiovascular comorbidities, (%)	11.4	-	58
Heart rate, mean ± SD/median [IQR], min^{-1}	78.5 ± 11.78	73 ± 13	78 [70-85]
Systolic blood pressure, mean ± SD/median [IQR], mmHg	123.4 ± 13.3	121 ± 14	112 [105-120]
eGFR, mean ± SD/median [IQR], $mL/min/1.73m^2$	61.92 ± 19.92	72.5 ± 22.3	67 [55-83]
Serum potassium, mean ± SD/median [IQR], mmol/L	4.27 ± 0.455	4.4 ± 0.7	4.4 [4.1-4.7]
NT-proBNP, median [IQR], pg/mL	At screening: 7310.4 ± 4991.26 At baseline: 4120.8 ± 3676.59	587 [214-2349]	At hospital admission: 4095 [2352-8160] At hospital discharge: 1390 [735-2835]
GDMT achieved			
RASi, (%)	97.2	97.0	100
TD RASi, (%)	51.8	-	73
≥ 50% TD RASi, (%)	82.3	55.0	93
βB, (%)	95.7	100.0	99

TD βB, (%)	45.0	-	57
≥ 50% TD βB, (%)	82.3	78.0	85
MRA, (%)	95.7	98.0	99
TD MRA, (%)	88.3	-	94
≥ 50% TD MRA, (%)	95.6	98.0	98
SGLT2i, (%)	-	93.0	99
TD SGLT2i, (%)	-	-	99
≥ 50% TD SGLT2i, (%)	-	92.0	99
TT, (%)	-	94.0	98
TD TT, (%)	-	-	51
≥ 50% TD TT, (%)	-	-	78
QT, (%)	-	88.0	98
TD QT, (%)	-	24.0	51
≥ 50% TD QT, (%)	-	44.0	78

DM: diabetes mellitus; eGFR: estimated glomerular filtration rate; GDMT: guideline-directed medical therapy; HFmrEF: heart failure with mildly reduced ejection fraction, HFpEF: heart failure with reserved ejection fraction; HFrEF: heart failure with reduced ejection fraction; IQR: interquartile range; LVEF: left ventricular ejection fraction; MRA: mineralocorticoid receptor antagonist; NT-proBNP: N-terminal pro-B type natriuretic peptide; QT: quadruple therapy; RASi: renin-angiotensin system inhibitor; RTP: rapid up-titration programme; SD: standard deviation; SGLT2i: sodium-glucose co-transporter 2 inhibitor; TD: target dose; TT: triple therapy; βB: beta-blocker.
